# Differences in Health Symptoms among Residents Living Near Illegal Dump Sites in Los Laureles Canyon, Tijuana, Mexico: A Cross Sectional Survey

**DOI:** 10.3390/ijerph110909532

**Published:** 2014-09-15

**Authors:** Wael K. Al-Delaimy, Catherine Wood Larsen, Keith Pezzoli

**Affiliations:** 1 Department of Family and Preventive Medicine, Division of Global Health, University of California, San Diego, 9500 Gilman Dr. #0628, La Jolla, CA 92093, USA; E-Mail: calarsen@ucsd.edu; 2 Urban Studies and Planning Program, University of California, San Diego, 9500 Gilman Dr., La Jolla, CA 92093, USA; E-Mail: kpezzoli@ucsd.edu

**Keywords:** cross-sectional survey, environmental, exposure, hazardous waste, Mexico, Superfund, symptoms

## Abstract

Living near landfills is a known health hazard prompting recognition of environmental injustice. The study aim was to compare self-reported symptoms of ill health among residents of four neighborhoods, living in haphazardly constructed settlements surrounded by illegal dumpsites in Tijuana, Mexico. One adult from each of 388 households located in Los Laureles Canyon were interviewed about demographics, health status, and symptoms. Distance from each residence to both the nearest dumpsite and the canyon bottom was assessed. The neighborhoods were selected from locations within the canyon, and varied with respect to proximity to dump sites. Residents of San Bernardo reported significantly higher frequencies of ill-health symptoms than the other neighborhoods, including extreme fatigue (OR 3.01 (95% CI 1.6–5.5)), skin problems/irritations (OR 2.73 (95% CI 1.3–5.9)), stomach discomfort (OR 2.47 (1.3–4.8)), eye irritation/tears (OR 2.02 (1.2–3.6)), and confusion/difficulty concentrating (OR 2.39 (1.2–4.8)). Proximity to dumpsites did not explain these results, that varied only slightly when adjusted for distance to nearest dumpsite or distance to the canyon bottom. Because San Bernardo has no paved roads, we hypothesize that dust and the toxicants it carries is a possible explanation for this difference. Studies are needed to further document this association and sources of toxicants.

## 1. Introduction

Rapid urbanization and industrial development that takes place without adequate infrastructure such as sewer systems, potable water, and proper hazardous waste disposal can put human health at risk and cause environmental degradation [1]. Along the 3145 km US-Mexico border, industrialization accelerated in 1964 with the initiation of the Border Industrialization Program. This was a bi-national agreement providing economic incentives for foreign-owned facilities in Mexico to export products back to the US [2], while increasing the demand for labor. This encouraged migration from all parts of Mexico and resulted in a population growth along the Mexican side of the border from 3,762,963 residents in 1950 to 13,246,991 residents in 1990 [3]. Since the mid-1990s, urbanization along the US-Mexico border continued its growth due to the North American Free Trade Agreement (NAFTA), characterized by the construction of manufacturing facilities in close proximity to worker housing lacking basic public infrastructure such as sewers, water, and paved streets [4,5,6]. The population of Tijuana, Mexico increased from 461,267 to 1.5 million residents between 1980 and 2010 [3], and it is currently the 5th largest city in Mexico. This growth pattern is expected to continue due to the continual demand for low skilled workers in the maquiladoras [1,7]. The maquiladoras are factories that produce a wide range of consumer goods for transnational corporations using raw materials imported mostly from the United States [8,9]. The 2010 population of the San Diego-Tijuana border region was 4.8 million, making it the largest bi-national metropolitan area shared between the United States and Mexico and the third largest bi-national metropolitan area in the world [3,10]. The environmental burdens of the border region’s haphazard development, are well documented [6,11], exposing residents to hazardous waste toxicants in the air, water, sewage, soil, and dust potentially causing health problems [2,7]. With the growth of the maquiladora industry, came a large surplus of hazardous waste [12]. For example, the U.S. owned maquiladora in Tijuana, “Metales y Derivados”, abandoned in 1994, was a lead recycling and smelter site that exposed nearby residents and workers to toxic levels of wind-blown lead particulates until its eventual clean-up in 2004 [13]. According to a 2004 report by Texas Center for Policy studies, only about 10% of Mexico’s hazardous waste receives proper treatment, while 50–80% of Mexico’s hazardous wastes are dumped illegally [14].

Los Laureles Canyon, a 4.6 square miles area and home to approximately 80,000 residents [15], is a watershed sub-basin of the bi-national Tijuana River Basin. This basin straddles the US-Mexico border, where water originating in Mexico drains north to the US, and eventually the Pacific Ocean. Urbanization fueled by the growth of maquiladoras and other industries has taken place mostly before any adequate public infrastructure was built (*i.e.*, there is deficit in paved roads, sewers, storm drainage, and other services). Residents have had to improvise and live without some basic public health necessities, often in conditions where they are exposed to hazardous environmental toxicants [14,16]. The informal human settlements that do not officially meet standard building codes, zoning, or public infrastructure regulations are known in Mexico as Colonias. The houses are makeshift structures, illegally built in the canyons and surrounding hills to be close to the maquiladoras and other industries, because residents cannot afford proper housing in regular neighborhoods in Tijuana [16]. During the rainy season, dangerous flash flood waters can wash away community roads, cars, structures, sediment, and debris [17,18]. Because local waste management has not kept pace, canyon residents are also affected by the illegal dumping of wastes generated by residents, business, hospitals, and factories [4,19].

Exposure to outdoor and indoor environmental contaminants can cause a variety of health symptoms [20], presenting as common medical ailments such as headaches, difficulty concentrating, rashes, and breathing problems [21,22]. Soil and dust have been identified as important pathways of toxicant exposure [23,24,25,26,27,28,29,30]. A study conducted in Chiapas (Mexico) measuring the levels of dichlorodiphenyltrichloroethane (DDT) and its metabolites in soil and indoor dust, found that DDT concentration in the dust sampled was much higher than the DDT concentration found in soil [31]. Further, toxicants such as heavy metals have been found to be more orally bio-accessible in dust than in soil [30]. Along the US-Mexico border region, toxic exposure and environmental contaminant studies have focused on lead [32,33] and polybrominated diphenyl ethers (PBDE) exposure [34]. Other border population environmental exposure studies have focused on characterizing airborne particle emissions [35,36,37,38], toxicants in urban storm water runoff [39,40], and drinking water quality [41].

The main aim of the study was to explore potential associations of living and environmental conditions, including proximity to dumpsites, with health symptoms. Specifically, we conducted a cross sectional survey to assess health and living conditions among the residents of three colonias (neighborhoods) located within Los Laureles Canyon, and one located along the ridge of the canyon. These four locations were chosen because they each varied in their juxtaposition in relation to closeness and number of illegal dumpsites. The objectives were to conduct an environmental needs assessment and epidemiological survey to characterize this population’s demographics, living conditions, and self-report health, as an exploratory study of environmental risk factors in relation to vicinity to dumpsites. We assessed the prevalence of perceived toxicant exposure symptoms of residents, according to their locations in the canyon. No such studies have been published about the health effects associated with living in these heavily contaminated canyons.

## 2. Methods

### 2.1. Study Population

Residents from four colonias were selected to participate in the survey. Three of the colonias, San Bernardo, Divinia Providencia, and Rancho Las Flores, are located within lower-lying areas of Los Laureles Canyon, whose residents are mostly a working class population. The fourth colonia, El Mirador, is located along the upper rim, above the canyon with more affluent residents than the other three colonias participating in the survey.

**Figure 1:**
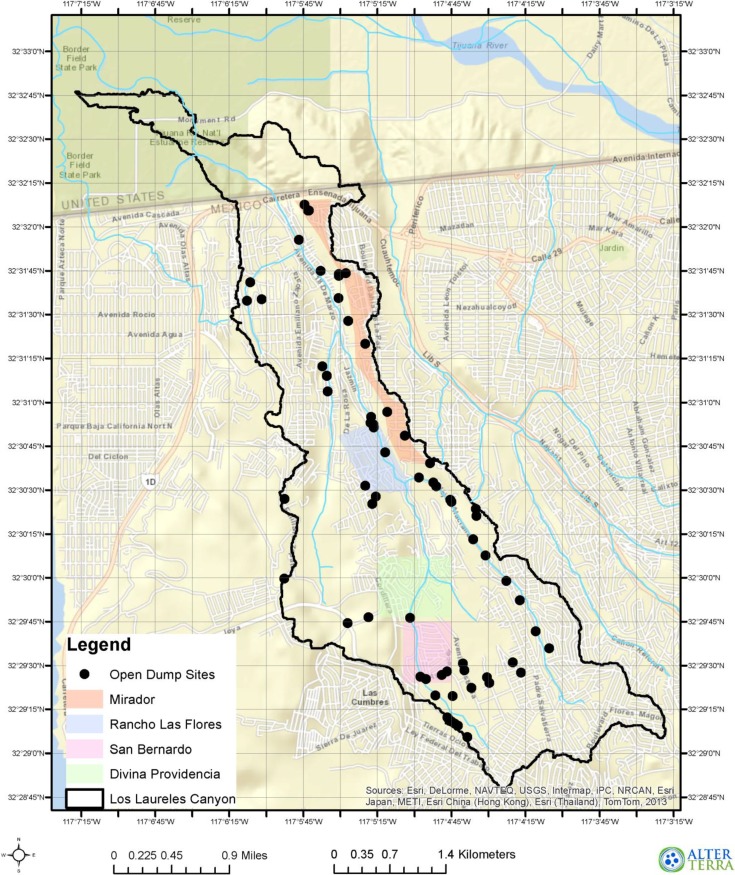
Map of Los Laureles Canyon including surveyed neighborhoods and dump sites. Figure image courtesy of Oscar Romo and Jennifer Hazard.

There are many illegal dump sites of varying sizes and waste composition throughout the canyon (at its base, on the slopes and along the rim, Figure 1). This has raised concerns among residents about possible exposure risk and negative health impacts. At least 10 dumpsites are located along the East rim of the canyon adjacent to El Mirador and five of these are along the canyon ridge above Rancho Las Flores. The contents of these dumpsites include construction debris, household waste, organic waste, old tires, and old appliances. In addition, there are six more dumpsites along the canyon bottom within or adjacent to Rancho Las Flores. Contents of these dumpsites include construction debris, old tires, animal waste, and soil. Two dumpsites are located on the west ridge of the canyon next to Divinia La Providencia, and one on the south edge of the colonia. These dumpsites contain a mixture of wastes. San Bernardo residents live in close proximity to 17 dump sites at the canyon’s most southern end and this is the only neighborhood with unpaved main roads and therefore more daily dusty conditions. Seven of these dumpsites are located along the southwestern ridge of the canyon, and consist of commercial waste, construction debris, ashes, and plastics. There are two dumpsites directly upstream from San Bernardo on the south side of the colonia consisting of hospital waste, commercial waste, and construction debris. Four dumpsites located along the south edge of San Bernardo consist of car parts, construction debris, plastics, foam, and mixed wastes. Lastly, there are four dumpsites adjacent to San Bernardo consisting of old pottery, industrial waste, household waste, construction debris, and organic waste [42].

### 2.2. Data Collection

Thirty one medical students from the Autonomous University of Baja California (UABC) School of Medicine and Psychology conducted the interviews by going door-to-door in each neighborhood. The students, led by medical school faculty, received 2 h of training by the local community organization Alter Terra that is partnering with us on this study. Starting from a centrally located intersection in each neighborhood, the interviewers were instructed to pair up, disperse in each direction, and approach every house for an interview. If there was no one home at that house, or if the person who answered refused to participate, the interviewers would go to the next house. All interviews were conducted in Spanish and took place between March and May of 2013. Interviewers explained to each participant that the purpose of the study was to assess their living conditions, and written informed consent was obtained. The residents were not aware that their responses would be analyzed within the context of adjacency to dumpsites. The study protocol and consent form was reviewed and approved by the University of California, San Diego Human Research Protection Program.

### 2.3. Cross Sectional Survey

Each survey was comprised of an in depth face-to-face 20 min interview consisting of 67 single-part and 21 multi-part questions, including five sections: (1) demographics; (2) living conditions; (3) perceived toxicant exposure symptoms, general health and lifestyle; (4) use and quality of various public services including medical; and (5) social programs. In this paper we focus on the results of the first three sections of the survey. The purpose of these questions were to address possible health impact of toxicants and the common confounders of such associations such as socioeconomic status represented by education and employment, and distance to sources of soil toxicants in dump sites or the bottom of the canyon.

### 2.4. Data Analysis

A total of 388 individuals, each representing a single household, were recruited. The response rate was 86.6%. Not all questions were answered by the participants, varying the sample sizes for each question. Participants were included in the data analysis if they answered at least 75% of questions in the questionnaire. The survey included questions about eleven perceived toxic exposure symptom variables among the households completing the surveys: (1) headaches; (2) confusion/difficulty concentrating; (3) memory problems; (4) ear/nose/throat irritations; (5) chest tightness; (6) difficulty breathing; (7) skin problems/irritations; (8) extreme fatigue; (9) stomach discomfort; (10) insomnia and (11) eye irritation/tears. Respondents were only aware that they were being asked the frequency of health symptoms, and were not made aware of the possible link between these symptoms and environmental exposure. These symptoms have been reported in previous studies and linked to toxicant exposure [21,22]. Arsenic exposure has been shown to cause headaches, sleeplessness, sleepiness, irritability, muscular fatigue [42], skin lesions [43], impaired lung function, and respiratory symptoms [44,45]. Lead exposure can cause abdominal pain [46,47], and impaired cognitive function [47,48,49]. Heavy metal dust and polycyclic aromatic hydrocarbon (PAH) dust exposure has been shown to cause symptoms such as dry cough, shortness of breath on exertion, and productive cough [50,51,52]. Respondents were asked to report the frequency of having each perceived toxic exposure symptom with six possible categorical responses. These responses ranged from “daily” to “very rarely”. Due to small number of participants in some of the categories of the variables we collected, we collapsed them for the purpose of data analyses. These categorical variables were dichotomized to the two responses of: (1) at least once a week; and (2) less often than once a week. For assessment of household education, we asked about the female head of household education level since most families are single mother families and this is a better reflection of the overall health education level of the household. The variable “female head of household education level”, with six categorical responses ranging from “no education” to “professional/teacher/doctorate degree” was collapsed into the three categories: (1) middle school and below; (2) high school; and (3) more than high school. The third variable, “employment status,” had the following six responses: (1) government employee; (2) laborer; (3) independent or office worker; (4) vender; (5) professional; (6) housewife; and (7) unemployed. These were collapsed into the following responses: (1) housewife/unemployed; (2) blue collar worker (laborer, vender); and (3) white collar worker (government employee, independent or office worker, and professional). In addition to a general question asking respondents if they lived or worked near a dumpsite, after the surveys were collected, we measured the distance of the middle of a respondent’s street to the bottom of the canyon and also to the nearest dumpsite was measured using Google Earth tools in order to better quantify the distance. We created two categorical variables, “Dumpsite Distance” and “Canyon Bottom Distance”. Each variable had the following four categories: (1) 100 m or less, (2) 101 to 200 m, (3) 201 to 300 m, and (4) More than 300 m. Extensive mapping of dumpsite locations was provided to us by our community partner Alter Terra [42].

Analysis was conducted using STATA version 10 (StataCorp LP, College Station, TX, USA). To measure differences in the means of continuous variables such as respondent’s age and number of years living in the colonias, the T-test and ANOVA tests were used. To test the hypothesis of differences between the colonias and the demographic and living condition categorical variables, Pearson’s chi-square and Fisher’s Exact tests were applied. If the statistical test resulted in a *p* value <0.05, the variable was selected for logistic regression and calculation of odds ratios to further explore relationships between the independent correlate of colonia and each of the eleven dichotomized perceived toxic exposure symptom variables. To adjust for the covariates of age, gender, education level, and employment status, multivariate logistic regression was employed, and odds ratios were calculated. A 95% confidence interval was used to determine significance. Lastly, the covariates of Dumpsite Distance and Canyon Bottom Distance were each added separately to the multivariate model to assess the effects of these variables on the Odds Ratios.

## 3. Results

Demographic characteristics and living conditions of each colonia are presented in Table 1. In El Mirador there was a lower percentage of female respondents (*p* = 0.028) and fewer households with children (*p* = 0.001) than in the other colonias. Respondents from San Bernardo were the youngest on average (*p* = 0.007), and had lived in their colonia the least amount of time (*p* < 0.001). Among all respondents 15.8% were smokers.

**Table 1:** Demographic characteristics and living conditions by colonia (n (%)) or (mean ±SD).

Variable	All Subjects	El Mirador	Las Flores	Divinia Providencia	San Bernardo	*p*-Value ^a^
Total	388	134	53	84	117	
Sex						0.028
Males	155 (40.2)	64 (47.8)	16 (30.2)	37 (45.1)	38 (32.5)	
Females	231 (59.8)	70 (52.2)	37 (69.8)	45 (54.9)	79 (67.5)	
Missing	2	0	0	2	0	
Age	40.7±14.3	43.7±14.3	38.4 ±13.4	41.5 ±13.8	37.8 ±14.4	0.007 ^c^
Years living in colonia	15.3 ±11.14	19.6 ± 12.7	13.0 ±7.3	14.2 ±8.5	12.4 ±11.1	<0.001 ^c^
Current smoker	61 (15.8)	24 (18.1)	7 (13.2)	12 (14.29)	18 (15.5)	0.820
Lives/works near dump ^d^	31 (8.4)	3 (2.4)	8 (15.1)	1 (1.3)	19 (17.1)	
Dumpsite distance ^d^ (N = 371)						
100 m or less	64 (17.3)	29 (23.2)	17 (32.7)	9 (11.3)	9 (7.9)	
101 to 200 m	144 (38.8)	40 (32.0)	35 (67.3)	34 (42.5)	35 (30.7)	
201 to 300 m	143 (38.5)	43 (34.4)	0 (0)	36 (45.0)	64 (56.1)	
More than 300 m	20 (5.4)	13 (10.4)	0 (0)	1 (1.3)	6 (5.3)	
More than one dumpsite within 300 m ^d^	63 (17.0)	0 (0)	5 (9.6)	0 (0)	58 (50.9)	
Has access to health care	354 (92.4)	120 (91.6)	48 (90.6)	77 (92.8)	109 (94)	
Has minor children living in home	244 (64.2)	70 (53.4)	44 (83.0)	57 (70.4)	73 (63.5)	0.001
Employment category						<0.001
White collar worker	98 (25.3)	50 (37.3)	5 (9.4)	20 (24.1)	23 (19.7)	
Blue collar	109 (28.1)	26 (19.4)	13 (24.5)	30 (36.1)	40 (34.2)	
Unemployed/housewife	180 (46.5)	58 (43.3)	35 (66.0)	33 (39.8)	54 (46.2)	
Female head of household education level ^d^						
Middle school or below	246 (64.1	47 (35.34)	46 (86.8)	66 (78.6)	87 (76.3)	
High school	83 (21.6)	40 (30.1)	5 (9.4)	15 (17.9)	23 (20.2)	
More than high school	55 (14.3)	46 (34.6)	2 (3.4)	3 (3.6)	4 (3.5)	
Primary household water source ^f^						
Hydrant outside home	144 (37.1)	39 (29.3)	18 (34.0)	33 (39.3)	54 (46.6)	0.041
Faucet inside the home	223 (57.7)	91 (68.4)	33 (62.26)	45 (53.6)	54 (46.6)	0.004
Has a sink in the home	316 (84.0)	122 (94.6)	35 (67.3)	69 (86.25)	90 (78.26)	<0.001
Home effluent drainage ^f^						
Public sewer system	281 (73.0)	127 (95.5)	18 (34.0)	47 (56.0)	89 (77.4)	<0.001
Septic tank	81 (21.0)	6 (4.5)	23 (43.4)	32 (38.1)	20 (17.4)	<0.001
Other ^d,e^	23 (6.0)	0 (0.0)	9 (17.0)	10 (12.0)	4 (3.5)	
Type of toilet in home						<0.001
No toilet	36 (9.4)	3 (2.3)	8 (15.7)	14 (16.7)	11 (9.7)	
Water with a bucket	52 (13.61)	4 (3.0)	15 (29.4)	14 (16.7)	19 (16.7)	
Flushing toilet	294 (77.0)	126 (94.7)	28 (54.9)	56 (66.7)	84 (73.7)	
Type of floor in home ^d^						
Dirt	24 (6.45)	1 (0.81)	6 (11.8)	9 (10.8)	8 (7.0)	
Cement	289 (77.7)	102 (82.3)	36 (70.6)	63 (75.9)	88 (77.2)	
Other	66 (17.84)	21 (17.9)	10 (19.6)	13 (16.1)	22 (19.3)	
Home cooking fuel ^d,f^						
Gas	362 (96.3)	127 (97.0)	52 (98.1)	79 (95.2)	104 (95.4)	0.76
Electricity	25 (6.7)	11 (8.4)	3 (5.7)	4 (4.8)	7 (6.4)	0.81^b^
Other ^d^	6 (1.6)	0	1 (1.9)	1 (1.2)	4 (3.7)	
Kitchen space in home ^d^						
No separate space	43 (11.2)	3 (2.3)	10 (18.9)	14 (16.7)	16 (14.0)	
Has a separate space	229 (59.8)	79 (59.9)	26 (49.1)	48 (57.1)	76 (66.7)	
Has a separate room	108 (28.2)	50 (37.9)	16 (30.2)	21 (25)	21 (18.4)	
Outside	3 (0.37)	1 (0.76)	1 (1.9)	0	1 (0.88)	
Type of home heating ^d,f^						
Gas	35 (9.1)	19 (14.3)	2 (3.8)	4 (4.8)	10 (8.8)	0.056 ^b^
Electricity	65 (16.9)	38 (28.6)	3 (5.7)	5 (6.0)	19 (16.7)	<0.001 ^b^
Other ^d^	5 (1.3)	1 (0.75)	0	2 (2.3)	2 (1.8)	
None	253 (65.9)	74 (55.7)	44 (83.0)	70 (83.3)	65 (57.0)	<0.001
Has a sink in the home	316 (84.04)	122 (94.57)	35 (67.31)	69 (86.25)	90 (78.26)	<0.001
Has a refrigerator	358 (92.3)	132 (98.5)	48 (90.6)	78 (92.9)	100 (85.5)	0.002
Has a cellular phone	332 (85.6)	120 (89.6)	45 (85.0)	68 (81.0)	99 (84.6)	0.35

^a^ *p*-values are for Pearson chi-square, unless noted otherwise; ^b^ *p*-Values are for Fisher’s Exact Test; ^c^ *p*-Values are for ANOVA, F-test; ^d^ Too few responses in cells to calculate *p*-value with chi-square or Fisher’s exact Test; ^e^ Other drainage of effluents includes canyon, river, street; ^f^ Subjects could respond yes or no for each item in this category.

There were statistically significant differences when comparing living conditions amongst the four colonias. Respondents from San Bernardo were least likely to have a water faucet inside their home (*p* = 0.004), and most likely to report their primary household water source as a public hydrant (*p* = 0.041). Respondents from Las Flores were least likely to report having a sink in their home (*p* < 0.001), plumbing for the drainage of effluent to the public sewer system (*p* < 0.001), and most likely to report having a septic tank (*p* < 0.001). Respondents from Las Flores and Divinia Providencia were most likely to report having no home heating (*p* < 0.001). Respondents from San Bernardo were more likely to live within 300 m of more than one dumpsite. Over 90% of respondents from each colonia reported having access to healthcare by one of the government sponsored programs, or a private pharmacy or clinic.

Table 2 presents the percentage of subjects who reported having each of the eleven perceived toxic exposure symptoms at least once a week. Respondents from Divinia Providencia and San Bernardo were significantly more likely to report having confusion or difficulty concentrating (*p* = 0.003), memory problems (*p* = 0.011), and ear/nose/ or throat irritations (*p* = 0.035) at least once a week. In addition, respondents from San Bernardo were most likely to report having skin problems/irritations (*p* = 0.032), extreme fatigue (*p* = 0.002), stomach discomfort (*p* = 0.003), and eye irritation/tears (*p* = 0.001) at least once a week. San Bernardo residents were most likely to report having a sick child at home within the past two weeks (*p* = 0.004), and to report that living in the canyon negatively affects their health (*p* = 0.001). Health questions regarding whether respondents had a history of having cancer, tuberculosis, diabetes, high blood pressure, or asthma did not vary significantly between the colonias (data not shown).

**Table 2:** Perceived toxicant exposure symptoms by colonia (n (%)).

Variable	El Mirador	Las Flores	Divinia Providencia	San Bernardo	*p*-Value ^a^
Number of subjects experiencing each symptom at least once a week					
Headache	34 (28.6)	18 (38.3)	31 (41.9)	45 (42.5)	0.12
Confusion/difficulty concentrating	3 (3.5)	5 (11.9)	13 (19.4)	18 (22.5)	0.001 ^b^
Memory problems	5 (5.7)	8 (18.6)	14 (22.2)	19 (22.1)	0.01
Ear, nose, or throat irritations	13 (12)	4 (8.5)	16 (22.5)	23 (23.5)	0.03 ^b^
Chest tightness ^b^	5 (5.7)	3 (7.0)	9 (13.9)	13 (16.9)	0.09 ^b^
Difficulty breathing	8 (9.4)	4 (9.5)	6 (9.4)	13 (16.5)	0.5 ^b^
Skin problems/irritations	4 (4.7)	4 (10.0)	7 (11.9)	15 (19.5)	0.03 ^b^
Extreme fatigue	10 (11.1)	5 (11.9)	12 (18.2)	28 (32.2)	0.002
Stomach discomfort ^b^	4 (4.7)	9 (20.5)	10 (19.4)	21 (25.3)	0.001 ^b^
Insomnia	18 (19.2)	6 (13.6)	17 (27.9)	24 (29.0)	0.14
Eye irritation/tears	18 (20.5)	7 (15.6)	21 (33.9)	30 (38.5)	0.01
Subjects having a sick child in the home within the last two weeks	6 (8.2)	11 (25.58)	16 (27.12)	26 (32.1)	0.004
Subjects who responded living in the ravine negatively affects health	44 (42.3)	35 (66.0)	41 (50)	72 (66.0)	0.001
How subjects rated their health ^c^					
Very good	18 (13.5)	0	8 (9.5)	6 (5.1)	
Good	81 (60.9)	23 (43.4)	35 (41.7)	52 (44.4)	
So-so	27 (20.3)	29 (54.7)	35 (41.7)	50 (42.7)	
Poor	6 (4.5)	1 (1.9)	5 (6.0)	8 (6.8)	
Very poor	1 (0.75)	0	1 (1.2)	1 (0.85)	

^a^ *p*-Values are for Pearson chi-square, except where specified; ^b^ *p*-Values are for Fisher’s exact test; ^c^ Too few responses in cells to calculate *p*-value with chi-square or Fisher’s exact Tests.

Given the clearly higher reporting of adverse health symptoms among San Bernardo residents, we further investigated the relationship between each perceived toxic exposure symptom and colonia by comparing responses from San Bernardo residents to residents from the other three colonias combined. Subjects in San Bernardo were significantly more likely to report experiencing confusion/difficulty concentrating (*p* = 0.012), ear, nose or throat irritations (*p* = 0.052), skin problems/irritation (*p* = 0.009), extreme fatigue (*p* < 0.001), stomach discomfort (*p* = 0.013), and eye irritation/tears (*p* = 0.013) at least once a week (Table 3). The odds ratios of reporting these perceived toxic exposure symptoms for San Bernardo residents *versus* non-San Bernardo residents were calculated by univariate logistic regression (Table 4). Subjects from San Bernardo were more than twice as likely than non-San Bernardo residents to report having skin problems/irritations (OR 2.73, 95% CI 1.3–5.9), stomach discomfort (OR 2.47, 95% CI 1.3–4.8), eye irritation/tears (OR 2.02, 95% CI 1.2–3.6), confusion/difficulty concentrating (OR 2.39, 95% CI 1.2–4.8), and three times more likely to report having extreme fatigue (OR 3.01, 95% CI 1.6–5.5) at least once a week. These results were consistent and changed minimally when multivariate logistic regression was applied to control for the demographic factors of age, gender, education level, and employment status (Table 4).

**Table 3:** Demographic characteristics, living conditions by colonia, and perceived toxicant exposure symptom San Bernardo compared to the other three colonias combined (n (%)) or (mean ± SD).

Variable	San Bernardo	Other 3 Colonias Combined	*p*-Value ^a^
	*n* = 117	*n* = 271	
Sex			
Males	38 (32.5)	117 (43.4)	0.042
Females	79 (67.5)	152 (56.5)	
Age	37.8 ±14.4	41.9 ± 14.1	0.0095 ^b^
Years living in colonia	12.4 ± 11.1	16.6 ± 11	0.0006 ^b^
Lives or works near dump	19 (17.1%)	12 (4.6%)	<0.001
Dumpsite distance ^d^ (N = 371)			<0.001 ^c^
100 m or less	9 (7.9)	55 (21.4)	
101 to 200 m	35 (30.7)	109 (42.4)	
201 to 300 m	64 (56.1)	79 (30.7)	
More than 300 m	6 (5.3)	14 (5.5)	
More than one dumpsite within 300 m ^d^	58 (50.9)	5 (2.0)	<0.001 ^c^
Has minor children in home	73 (63.5)	171 (64.5)	0.844
Female head of household education level			<0.001 ^c^
Middle school and below	87 (76.3)	159 (58.9)	
High School	23 (20.2)	60 (22.2)	
More than high school	4 (3.5)	51 (18.9)	
Employment Status			0.117
White collar worker	23 (20.0)	75 (27.8	
Blue collar	40 (39.2)	69 (25.6)	
Unemployed or housewife	54 (46.2)	126 (46.7)	
Has a sink in the home	90 (78.26)	226 (86.6)	0.042
Has a flushing toilet in the home	84 (73.7)	210 (78.4)	0.321
Has no home heating	65 (57.0)	188 (69.6)	0.017
Kitchen is a separate room	21 (18.4)	87 (32.3)	0.006
Principal source of domestic water ^d^			
Hydrant	54 (46.6)	90 (33.3)	0.014
Faucet inside the house	54 (46.55)	169 (62.6)	0.003
Perceived toxicant exposure symptom			
Headache	45 (42.5)	83 (34.6)	0.162
Confusion/Difficulty Concentrating	18 (22.5)	21 (10.8)	0.012
Memory Problems	19 (22.0)	27 (13.9)	0.089
Ear, nose, or throat irritations	23 (23.5)	33 (14.6)	0.052
Chest tightness	13 (16.9)	17 (8.7)	0.051
Difficulty Breathing	13 (16.5)	18 (9.4)	0.099
Skin problems/irritations	15 (19.5)	15 (8.2)	0.009
Extreme fatigue	28 (32.2)	27 (13.6)	<0.001
Stomach discomfort	21 (25.3)	23 (12.0)	0.006
Insomnia	24 (28.9	41 (20.6)	0.131
Eye irritation/tears	30 (38.5)	46 (23.6)	0.013

^a^ *p*-Values are for chi square test except where specified; ^b^ Two sample ttest; ^c^ Fishers Exact Test; ^d^ This specific dumpsite information was calculated using Google Earth tools.

**Table 4:** Univariate and multivariate logistic regression models for risk factor of living in San Bernardo to predict having each perceived toxicant exposure symptom at least once a week.

Predictor Variable	OR for Each Symptom (95% CI)									
	Skin Problems/Irritations		Extreme Fatigue		Stomach Discomforts		Eye Irritation/Tears		Confusion/Difficulty Concentrating	
	Unadjusted	Adjusted ^a^	Unadjusted	Adjusted ^a^	Unadjusted	Adjusted ^a^	Unadjusted	Adjusted ^a^	Unadjusted	Adjusted ^a^
Living in	2.73	2.94	3.01	3.06	2.47	2.46	2.02	2.06	2.39	2.09
San Bernardo	(1.3–5.9) *	(1.3–6.8) *	(1.6–5.5) ***	(1.6–5.9) ***	(1.3–4.8) **	(1.2–5.0) *	(1.2–3.6) **	(1.1–3.8) *	(1.2–4.8) *	(1.0–4.4) *

* *p* < 0.05; ** *p* < 0.01; *** *p* < 0.001; ^a^ Adjusted for age, sex, education level, employment category.

We explored the effects of distance from dumpsite and distance from the bottom of the canyon for each neighborhood and added them separately to the multivariate models but the results were not changed substantially (data not shown). Adding the categorical variable “Dumpsite Distance” to the multivariate logistic regression slightly increased the OR’s for “Skin irritations”, and “Stomach discomfort”, and slightly decreased the OR for “Eye irritation”, “Extreme Fatigue”, and “Memory problems/confusion”. “Dumpsite Distance” was then removed, and when “Canyon Bottom Distance” was added to the multivariate logistic regression model, the OR decreased slightly for those symptoms.

## 4. Discussion

Our study is the first large scale investigation aimed at characterizing the demographics, living conditions, and perceived toxic exposure symptoms of the residents living in makeshift housing and poor environmental conditions in Los Laureles Canyon. Our results demonstrated that living conditions in each colonia varied and the San Bernardo residents reported having five of eleven perceived toxic exposure symptoms at least once a week 2 to 3 times more often than the other three colonias combined. They are also more likely to report having a sick child in the home within the past two weeks, and that living in the canyon had a negative effect on their health. Our results clearly point to one neighborhood and its residents suffering the most frequent symptoms of ill-health. Our initial hypothesis to explain these differences as a function of decreasing distance between the respondents’ home and dumpsites or canyon bottom was not conclusive. By controlling for “Dumpsite Distance,” OR’s for “Skin irritations”, and “Stomach discomfort”, and slightly increased, while ORs for “Eye irritation”, “Extreme Fatigue”, and “Memory problems/confusion” slightly decreased. This indicates that “Dumpsite Distance” may be a contributing factor with regard to “Skin irritations”, and “Stomach discomfort” and less of a contributing factor to “Eye irritation”, “Extreme Fatigue”, and “Memory problems/confusion”. When we controlled for “Canyon Bottom Distance” ORs for these symptoms slightly decreased, indicating that location within the canyon may also be a factor. If distance to the nearest dumpsite or distance to the canyon bottom were the main contributing factors in the positive associations between Colonia San Bernardo and health symptoms, by controlling for these factors, one would expect the ORs to significantly decrease. With a larger sample size, we may have been able to demonstrate these relationships more conclusively. This was a descriptive study and if the variable “Dumpsite Distance” or “Canyon Bottom Distance” effect is small, a larger sample size would better detect this small difference [53]. Topographical differences between each colonia and the surrounding dumpsites may also be a factor. For example, if a colonia is located downwind or downstream from a dumpsite, the dumpsite proximity may affect residents differently than if the colonia is located upstream or upwind from a dumpsite, also indicating location within the canyon may be a contributing factor. Number and contents of dumpsite may also be contributing factors. Other studies have found spatial correlations of health symptoms of living near dump sites including an increased prevalence of self-reported eye irritation [54,55,56,57], skin rashes [54,55,56,57,58], abdominal pain [56], gastrointestinal symptoms [55,57,59], respiratory conditions such as wheezing and asthma [54,57,59,60,61], neurologic symptoms [62], muscle weakness [54], and fatigue [59]. The main limitations with these studies is that they rely on self-report and not on measures of direct exposure [63].

According to our survey data we cannot conclude that the close proximity to dumpsites are what is causing San Bernardo residents to report more ill health than the other colonias. Hypothesizing disease causation using spatial analysis is challenging due to the many possible etiological factors contributing to the onset of disease [64]. Without a measure of direct exposure, other environmental factors cannot be ruled out.

By reviewing the history of that specific neighborhood, it was found that before the houses started being built in 2004, the developer of San Bernardo scraped the native topsoil to form building lots. Residents bought lots and started building their own makeshift homes before any infrastructure such as paved roads and sewer, services were in place. This practice of removing all native vegetation has created very dusty conditions for this neighborhood [16]. Studies have shown land surface characteristics, such as vegetation, topography, surface winds, and soil wetness are important factors in controlling dust emissions [65,66], and those living near unpaved roads can have an increased exposure to unsafe levels of dust [67]. On the main road into the community, women and children walk along side moving vehicles on daily basis breathing and ingesting the dust from the disturbed soil near the bottom of the canyon, getting covered with dust on their skin, hair, and the insides their homes. Climate models of this region project a decrease in rainfall leading to dryer conditions, leading to dustier conditions in the future [68]. In contrast, residents from the other colonias live with much less dust, where houses were built without a mass removal of topsoil.

Prevalence of wheezing and atopic symptoms have been partly explained by housing and indoor environmental conditions in other populations [36,69], and house dust has been identified as an important pathway of toxic exposure. House dust may absorb pollutants released from activities and materials within a home or attahced to particles from outside the home [23,30]. Studies have found higher concentrations of toxicants in dust compared to soil [30,31].

In addition to dusty conditions, living with animals in San Bernardo was a common observation. This leads to further exposure from eating eggs and meat or drinking milk from these animals. It was observed that residents from the other colonias live with fewer domestic animals because of limited space on the side or upper part of the canyon compared to the bottom of the canyon.

Even though our findings with regard to close proximity to dumpsites was inconclusive, previous studies have shown the negative effects of toxic waste sites on the environment and human health [70] including an increase in respiratory symptoms, reduced lung function [71,72], and spatial associations of an increased risk of certain birth defects [73,74,75,76,77,78], low birth weight [79], and an increased incidence of miscarriages [80].

The large number of such dump sites and the thousands of residents living near them in make shift houses under extremely dusty conditions is a unique case of environmental injustice due to the sheer magnitude of those affected, when compared to what is documented in the literature from the US and the more developed countries [81,82].

Findings that residents in different colonias experience a different amount of perceived toxicant exposure symptoms supports the hypothesis that the living environments of the four colonias studied may be different with respect to exposure to hazardous environmental toxicants. More specifically, our findings suggest that the living conditions in San Bernardo are related to reporting more symptoms than the other colonias. Their symptoms of skin problems/irritations, stomach discomforts, eye irritation/tears, confusion/difficulty concentrating, and extreme fatigue are consistent with previous studies reporting symptoms that are present when one is exposed to environmental toxicants [21,22,83,84,85].

The study does have limitations. This is not a case control study. However, having a control population with no exposure and located outside the canyon, would have other more significant biases of SES, health care access, and non-environmental factors. This study was dependent on self-reported symptoms of residents, unconfirmed by a physician. The link between the living environment and evidence of perceived toxic exposure is therefore inferred ecologically. The consistency of results for the San Bernardo colonia compared to all other colonias and after adjusting for multiple covariates is somewhat reassuring in support of this association between living in this neighborhood and reported symptoms. Further, the reporting by San Bernardo residents that living in the canyon is affecting their health at a higher frequency than that from other areas is additional internal validity regarding adverse health impact from the environment on their health. Direct evidence of this relationship is needed by analyzing the drinking water, soil, dust or air quality in each colonia to identify toxicants causing such health symptoms among Los Laureles Canyon residents [23,27,28,31]. The connection between environmental exposure to toxic substances and symptoms could be further realized by the use of biomarkers. Biomarkers of blood, urine, breast milk, and toenails have been used in studies to detect environmental exposures of toxic substances [34,84,86,87,88,89,90].

Another limitation was that we used convenient sampling rather than random sampling. This introduces the possibility of selection bias. Random sampling, such as calling random phone numbers out of a phone book, was not logistically possible, because many of these residents have no landline phone connected to their address. Those who wished not to participate or who were not at home when the survey was conducted may have had different responses than those who participated. However, the fact that our response rate was very high and we chose every household in the selected streets of the 4 neighborhoods is likely to limit selection bias. The information collected was self-report of symptoms and not actual diseases diagnosed by a physician. Self-report data is sometimes subjective because it is dependent on a person’s memory as well as their perception of the symptom. Confirming the residents’ symptoms with health outcome data from the local health clinics is not feasible, given the nature of Tijuana’s healthcare system where there isn’t a specific clinic or hospital in the vicinity where Los Laureles Canyon residents receive medical treatment.

Lastly the potential confounding factor of socio-economic status was based on education and occupation, which has been used to reflect socioeconomic status in other health outcome research [91]. We found no significant differences in the self-reporting of common chronic diseases reported between groups. Not including household income might lead to residual confounding among those with higher education but low income employment. However, most canyon residents are not wealthy and only 14% had education higher than high school,

## 5. Conclusions

Our findings suggest that living conditions in Los Laureles Canyon are affecting residents’ health. This is a case of global environmental injustice resulting from expansion of multi-national industries, illegal dumping, poverty and poor housing in areas that lack proper public infrastructure. Specifically, those living in San Bernardo are reporting more perceived toxicant exposure symptoms. These symptoms includes confusion/difficulty concentrating, ear, nose or throat irritations, skin problems/irritations, extreme fatigue, stomach discomfort, and eye irritation/tears. We could not verify that proximity to sources of toxicants in soil can explain these symptoms among residents of this specific neighborhood, but we believe dust and the toxicants it carries might be one possible explanation and the factor specifically affecting this neighborhood. This exploratory study indicates that more studies are needed to further investigate which toxicants residents are being exposed to and the source of that exposure. This study does provide initial evidence about reported symptoms in this population to support larger and more complex epidemiologic designs. Such studies will be able to document the source of toxicants and how soil toxicants may lead to exposure of grossly underserved populations at the global level and potential plans to prevent future exposure through urban planning remediation effort as basic as paving the roads in such areas.
